# The Parietal Cortex in Sensemaking: The Dissociation of Multiple Types of Spatial Information

**DOI:** 10.1155/2013/152073

**Published:** 2013-04-21

**Authors:** Yanlong Sun, Hongbin Wang

**Affiliations:** University of Texas Health Science Center at Houston, 7000 Fannin, Suite 600, Houston, TX 77030, USA

## Abstract

According to the data-frame theory, sensemaking is a macrocognitive process in which people try to make sense of or explain their observations by processing a number of explanatory structures called frames until the observations and frames become congruent. During the sensemaking process, the parietal cortex has been implicated in various cognitive tasks for the functions related to spatial and temporal information processing, mathematical thinking, and spatial attention. In particular, the parietal cortex plays important roles by extracting multiple representations of magnitudes at the early stages of perceptual analysis. By a series of neural network simulations, we demonstrate that the dissociation of different types of spatial information can start early with a rather similar structure (i.e., sensitivity on a common metric), but accurate representations require specific goal-directed top-down controls due to the interference in selective attention. Our results suggest that the roles of the parietal cortex rely on the hierarchical organization of multiple spatial representations and their interactions. The dissociation and interference between different types of spatial information are essentially the result of the competition at different levels of abstraction.

## 1. Introduction

Sensemaking is a complex cognitive activity in which people make sense of or explain their experience or observations. Sensemaking is ubiquitous in humans' everyday life. Examples of sensemaking include medical diagnosis, scientific discovery, and intelligence analysis. Though it is plausible to argue that the core of sensemaking is abduction (a reasoning process that generates and evaluates explanations for data that are sparse, noisy, and uncertain), there is no doubt that sensemaking is not a primitive neurocognitive process. Rather, sensemaking is comprised of a collection of more fundamental cognitive processes (e.g., perception, attention, learning, memory, and decision making) working together, and certainly involves a group of brain systems from posterior regions to the prefrontal cortex.

According to the data-frame theory of sensemaking, people possess a number of explanatory structures, called frames, in which people try to fit the data into a frame and fit a frame around the data, until the data and frame become congruent [1–3]. Sensemaking is called a macrocognitive process in that it involves complex data-frame interactions (e.g., frames shape, define data, data recognize, and mandate frames), and therefore requires coordinated activities from multiple cognitive processes/systems, including attention, learning, memory, reasoning, and decision making. Whereas many different types of integrative processing models exist, the data-frame theory brings clearly into focus the emergence of the explanatory structures and the opportunity of learning in terms of extracting statistical regularities from the environment [4]. Such an approach makes the theory particularly appealing when the task environment is complex and people have to make decisions in the presence of multiple cues with a great deal of uncertainty.

**Figure 1:**
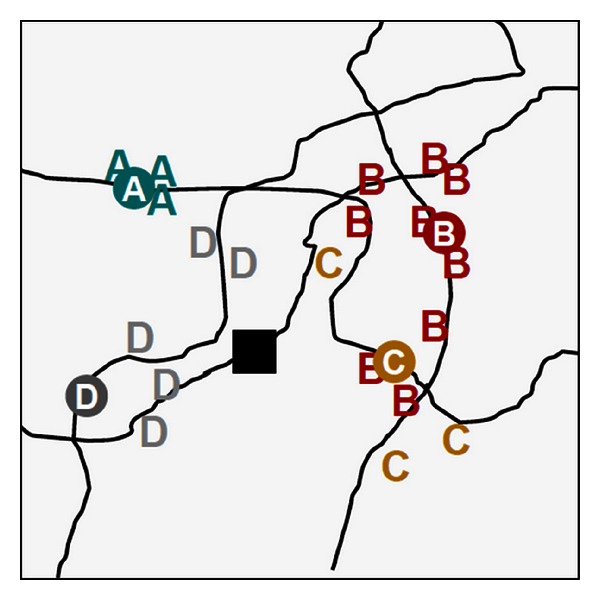
A typical scene in the COIN-AHA tasks. Attacks from individual enemy groups (labeled by “A”, “B”, “C”, and “D” in different colors) are distributed along a road network. Subjects first need to estimate the radius and the center of gravity of the attacks from each enemy group. When a new attack occurs at the point of interest (POI, represented by a black square), subjects are asked to report the likelihood of each enemy group responsible for such an attack.

Figure 1 describes a counter-insurgency surveillance example (hereafter COIN-AHA problem), in which an analyst is faced with a map that records the attacks from multiple enemy groups in the past. (For detailed modeling problems, see MITRE's Technical Report, In Press). The task is to estimate which enemy group would be more likely to be responsible for the new attack at the provided location (point of interest, POI). This task is clearly a sensemaking task. In particular, since the task stimuli are presented in a spatial environment, for effective sensemaking, different types of spatial properties of the environment would have to be acquired in the first place. For example, how many attacks have been carried out by each group (e.g., counting the number of objects from the visual inputs)? How large is the area that each group's attacks cover (e.g., perception of dispersion or size)? How close is the new attack to each group's active area (e.g., estimation of distance)?

Among the multiple cognitive steps in decision making, the parietal cortex (PC) has been implicated in various tasks for the functions related to spatial and temporal information processing, mathematical thinking, and spatial attention [5–8]. In the context of understanding the sensemaking processing in the COIN-AHA tasks, all these functions are certainly relevant. In this paper, we report a computational model to simulate the various functions of the parietal cortex in sensemaking (hence the PC module). In doing so, we hope to provide an integrated theory of the parietal cortex in spatial-temporal processing. 

## 2. Value Representation in the Parietal Cortex

The central theme in modeling the parietal cortex in the COIN-AHA tasks is the estimation, representation, and integration of values based on the magnitudes of various spatial properties such as numerosity, group center, distance, and probability. Before we dive into the modeling details, we first discuss the unique role of the parietal cortex in value representation in the broader context of judgment and decision making. 

First of all, in most accounts of decision theories, value representation is considered as the essential component in the decision-making process. To an extreme extent, the entire process of decision making is about value representations (e.g., [9]). Vlaev et al. [10] summarized three types of decision theories. The approach of “Type I”, value-first decision making, is based on independent and absolute value scales (e.g., [11]). “Type II”, comparison-based decision making with value computation, is based on comparison of values where subjective magnitude representations are context dependent (e.g., [12]). “Type III”, comparison-based decision making without value computation, has no explicit psychoeconomic scales, and decisions can be reached at by binary comparison, for example, by the “priority heuristic” (e.g., [13]). Despite the different flavors, all the three types of the decision theories have to rely on some form of value representations. The difference is only on the specific forms and stages of value representation in decision making: for example, whether the value representation stably leads to a decision (Type I) or is modulated by contextual information (Type II), or whether the value is on a cardinal scale (e.g., number or magnitude-like in Types I and II) or an ordinal scale (e.g., binary comparison in Type III). Neurologically, we are interested in the neuronal correlates of values in decision making in addition to the value representation itself. It has been reported that the neuronal correlates of various types of values exist in numerous regions, such as the orbitofrontal cortex, parietal cortex, posterior cingulated cortex, dorsolateral prefrontal cortex, premotor cortex, and frontal eye fields (for reviews, see, [9, 14]). Thus, it is critical to examine different types of value representations depending on the purpose and domain of the brain function.

Compared with other brain regions, the parietal cortex plays a unique role in transforming the spatial and temporal information from the environment, such as time, distance, speed, size, and numerosity, into magnitude-like value representations [5, 15]. Neuroanatomically, the parietal cortex receives projections from multiple sensory modalities, including visual, somatosensory, and auditory. In addition, it receives inputs from the subcortical collicular pathway, which consists of the superior colliculus and the pulvinar and is thought to be closely related to spatial orienting and eye movement control [6, 16, 17]. Most significantly, the parietal cortex has been identified as part of the dorsal “where” pathway [18]. It has been indicated that there is *a common metric* of time, space, and quantity representations residing in the parietal cortex because of the need to learn about the environment through motor interactions and to encode relevant variables for action [5, 15]. This pattern of cortical connections makes the parietal cortex an ideal system for integrating and extracting spatial information from multiple modality-specific and unstable sensory channels and achieving supramodal and more stable spatial representations.

Although the encoding of values could be relevant in all stages of decision making and exhibit neuronal correlates in numerous brain regions, the value representation in the parietal cortex is unique in that it is confined by its *proximity* and *specificity* [7]. For example, fMRI studies revealed that when participants were instructed to compare number, size, and luminance, the activation of the left and right intraparietal sulci (IPS) shows a tight correlation with the behavioral-distance effect [7, 19]. Whereas hippocampal and parahippocampal regions are clearly involved in spatial cognition, they do not possess the close proximity of spatial and numerical representations as the parietal cortex does. Although frontal regions are involved in both spatial and numerical tasks, parietal activations are related to a more restricted set of cognitive processes. Such specificity probably is most evident in the comparison between the orbitofrontal cortex (OFC) and the lateral intraparietal area (LIP). In general, the values represented by LIP neurons are more subject to modulation of responses encoding the spatial properties of the visual stimuli [9]. Although there is evidence that neurons in LIP are sensitive to probabilistic classification, it seems that such a sensitivity is limited to the simple integration of visual properties (e.g., combination of shapes) [20]. In contrast, neurons in OFC represent the value of goods per se (probabilities, rewards, etc.), independently of how goods are visually presented [9]. Crucially, the bilateral horizontal segment of the intraparietal sulci (HIPS) that are consistently activated in arithmetical tasks in humans roughly coincides with the putative human ventral intraparietal area (VIP), and such an overlap between comparison processes and spatial networks in the IPS is believed to account for the behavioral interactions between representations of number and space [7]. In sum, the parietal cortex, and the IPS in particular, might be the first cortical stage that extracts visual numerical information from visual inputs [8].

Another aspect of the specificity in the parietal cortex's spatial processing comes from the selection of frames of references (FOR). While spatial representations prior to the parietal cortex are typically retinotopic, spatial representations in the parietal cortex have been transformed and are generally egocentric. In putative human homologues of macaque IPS regions, LIP represents target position in an eye-centered frame of reference and is involved in spatial updating. Ventral intraparietal (VIP) represents targets in a head-centered frame of reference, and anterior intraparietal (AIP) represents space in hand-centered coordinates [7]. According to theory of frame of reference-based maps of salience (FORMS), the parietal cortex subserves spatial representations using a range of egocentric frames of references (e.g., eye centered, hand centered, and body centered, etc.) so as to allow rapid actions [21–23]. In addition, intrinsic representations, which represent between-object relations using a world-centered frame of reference but often involve some degree of perspective taking, are also encoded in parietal cortex, especially the posterior parietal cortex [24]. Furthermore, the values encoded by the parietal cortex tend to be at the approximate level rather than exact. For example, it has been indicated that topological comparison and approximate metrics are encoded within the parietal cortex, and exact spatial metrics are encoded in hippocampus [25]. 

**Figure 2:**
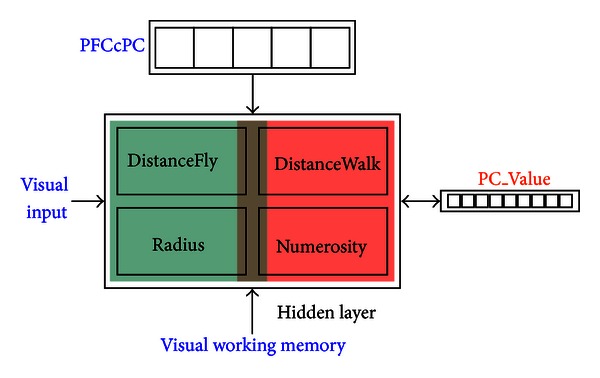
A schematic depiction of the metric estimates in the PC module. A common hidden layer takes inputs from both visual field and visual working memory, and outputs a magnitude value as the “PC value”. There are two basic metrics being encoded on the hidden layer: numerosity (in red area, including the number of group attacks and the walking distance on a road segment) and size (in green area, including the radius of group attacks and the flying distance between the group center and POI). The overlap of red and green areas represents the possible overlapped functionality of numerosity and size. The role of PFCcPC (PFC controls PC) is to provide an “attentional prioritization” by enhancing the contrast and specialization on the hidden layer. At any time, only one of the units on PFCcPC is active and projected onto the corresponding section on the Hidden layer. As a result, that section is more active than other sections and more likely to win the competition.

In accordance with theories described above, we have designated the PC module to be responsible for (1) extracting and representing relevant spatial information (i.e., providing relevant data from frame-matching such as radius, group center and two types of distances); (2) providing mechanisms for shifting attention during the process (i.e., defining and shaping data collection through both top-down and bottom-up modulations). Specifically, the PC module is responsible for processing the following spatial information (see Figures 1 and 2):

1. estimating the group center, the centroid of a cluster of attacks from a particular enemy group;
2. estimating the dispersion (“Radius”), the two-to-one radius that spatially covers two-thirds of the attacks from a known enemy group;
3. estimating two types of distances between the enemy group center and the point of interest (POI): “DistanceFly” represents the Euclidean distance (as crow flies) and “DistanceWalk” represents the length of the road segment (as cow walks);
4. estimating the number of attacks from each enemy group (“Numerosity”), which will later lead to the base rate comparison (i.e., the percentage each enemy group takes in the total number of attacks).

In the following, we discuss the implementation steps and the corresponding psychoneurological justifications in detail.

## 3. Inputs: Perceptual Grouping and Segmentation

The first step in modeling the parietal cortex functions in the COIN-AHA tasks is to group the various visual representations (e.g., individual attacks, POI, road) onto different input layers. On the one hand, our modeling focus is on the higher-order functions of value representation rather than the low-level visual processing. On the other hand, the encoding of values in the parietal cortex is heavily driven by the spatial and temporal properties of the visual inputs. To strike a balance, we made several simplifications in organizing the input layers to the PC module.

**Figure 3:**
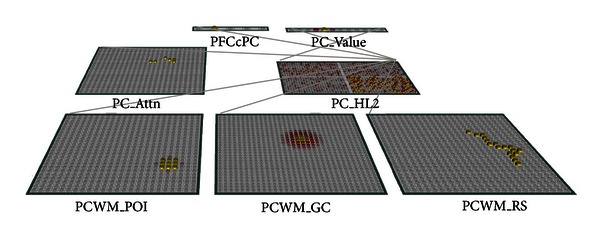
The parietal cortex module for computing target values on Numerosity, Radius, DistanceFly, and DistanceWalk (represented on the target layer “PC_Value”). The hidden layer “PC_HL2” is “sliced” into 4 groups, each responsible for a different type of target values. The competition on PC_HL2 is achieved by a kWTA function (k-Winners-Take-all, [26]), which is a combination of within-group inhibition (only the most active units within a group can contribute as the layer output) and entire-layer inhibition (units within a relatively weaker group are more likely to be inhibited). Such a function allows both of the dissociation and interference between different types of target computations. In the bottom-up information flow, input for both Numerosity and Radius is represented on PC_Attn (group attacks on visual field); Input for DistanceFly is based on a direct comparison of PCWM_POI (point of interest) and PCWM_GC (group center). Input for DistanceWalk is represented on PCWM_RS (road segment between POI and group center). The top-down control from layer PFCcPC represents 4 types of magnitude computation (Numerosity, Radius, DistanceFly, DistanceWalk) (the fifth unit is tentatively reserved for topological comparison). At any time, only one type of the magnitude values is available at the output level. The example shown here illustrates the case when the top-down demand is to compute Numerosity (second unit on PFCcPC), such that the bottom-right section on PC_HL2 is more likely to win over other sections in kWTA inhibition.

To represent the multiple attacks from enemy groups (e.g., attacks labels “A,” “B,” “C,” and “D” in Figure 1) within the parietal cortex, our modeling approach is to represent the multiple attacks from a single enemy group as a whole on the visual input layer (“PC_Attn”, see Figure 3), separated (segmented) from the attacks from other enemy groups. Then, both *numerosity* and *radius* can be computed based on PC_Attn. Next, the group center is computed as the *center of gravity* (i.e., arithmetic means of *x* and *y* coordinates of individual attacks) and represented on layer “PCWM_GC.” The point of interest (POI) is represented on a separate input layer “PCWM_POI” (with lateral activations such that an object is displayed as a Gaussian bump). Then, DistanceFly (the distance “as crow flies”) is computed as the Euclidian distance between the group center and POI. To compute DistanceWalk (the distance “as cow walks”), we represent the road segment between the group center and the POI on the input layer “PCWM_RS”. Then, the estimation of the walking distance is in effect to estimate the length of a curved line segment, which is equivalent to numerosity counting based on the number of activated pixels on PCWM_RS, regardless the topographic distribution of individual pixels.

It is noted that in the current model, we have avoided the problem of finding the shortest path. Instead, we focus on the problem of length estimation when a road segment is explicitly provided (i.e., the walking distance). In representing the road segment as a separate visual input, our justification is that a curved line segment can be recognized and maintained as a single visual input component. Ungerleider and Bell [17] suggest that in identifying and discriminating the primitive “geons,” neuronal selectivity progresses from simple line segments (in V1) to simple curves (in V2), to complex curves or combination of curves (in V4 and posterior IT cortex). In addition, it has been suggested that attention operates on object-centered as well as on location-based representations in that two connected objects (e.g., a barbell) may be represented as a single continuous object [27].

**Figure 4:**
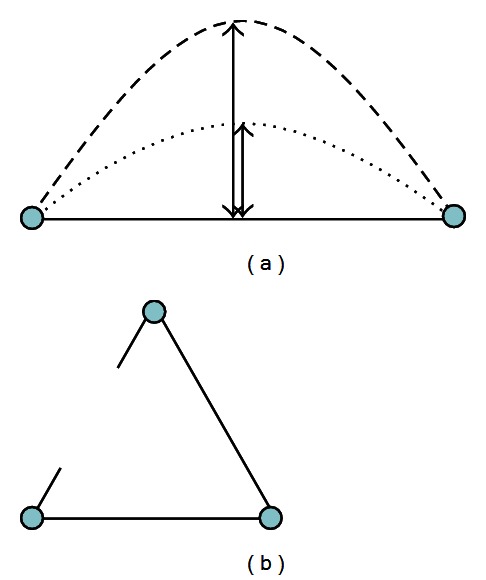
Distance adjustments by curvature and connectedness. By representing the road segment on a grid, the adjustment by curvature and connectedness is transformed into the task of numerosity estimation. For example, a more curvy (greater sagitta) road segment presents more active pixels on the grid (a), and a disconnected object pair requires additional routes to be connected and results in more active pixels on the grid (b).

Apparently, estimating the walking distance between two objects will be affected by the curvature (the curves on the road) and connectedness (whether two objects are connected by the road). Regarding the curvature (Figure 4(a)), it has been suggested that the “sagitta” provides the best cue in accounting for the discrimination of pairs of long-duration, curved-line stimuli, over a range of one- and two-dimensional transformations, and the contour curvature was coded in terms of just two or three curvature categories, depending on curved-line orientation [28]. Regarding the connectedness (Figure 4(b)), Sun and Wang [21] found that object pairs connected or anchored to the same landmarks are easier to recall than those anchored to different landmarks. Together, these studies suggest that people are to a certain extent sensitive to the variations in curvature and connectedness. Then, by representing the road segment on a single layer, the curvature and connectedness are in effect implicitly encoded in the visual input. For example, a curvy road segment would be longer than a straight one, and two points directly connected by the same road would be closer than connected by different roads because of more curves. Then, estimating the walking distance along the road effectively becomes a task of numerosity estimation (i.e., counting the number of active units on the line segment), resulting in a nonverbal representation of magnitude and number sense housed in IPS [7, 29].

It is noted that our method of representing various inputs to the PC module is mostly guided by the principles of *selective attention*. In particular, the PC module may receive multiple perceptual inputs in parallel from both direct visual input (layer “PC_Attn”) and visual working memory (“PCWM_” layers), but the total number of input layers is limited. This is due to the consideration that when the computation of target values requires selective attention (e.g., paying attention to one particular enemy group), it generally suffers a bottleneck that poses more strict limitations on the processing capacity (e.g., [30, 31]). In addition, we also considered the constraints to display resolutions on the input layers. It appears that the superior intraparietal sulcus (SIPS) could be the candidate for providing inputs from visual working memory, with a high resolution but a limited number of slots [32]. Also note that at the current stage of modeling, the assignment of whether a particular input is directly from visual field or from visual working memory is rather arbitrary. In reality, it is likely that the assignment will be dependent on the temporal sequences of visual stimuli or on specific strategy usages by individual subjects.

**Figure 5:**
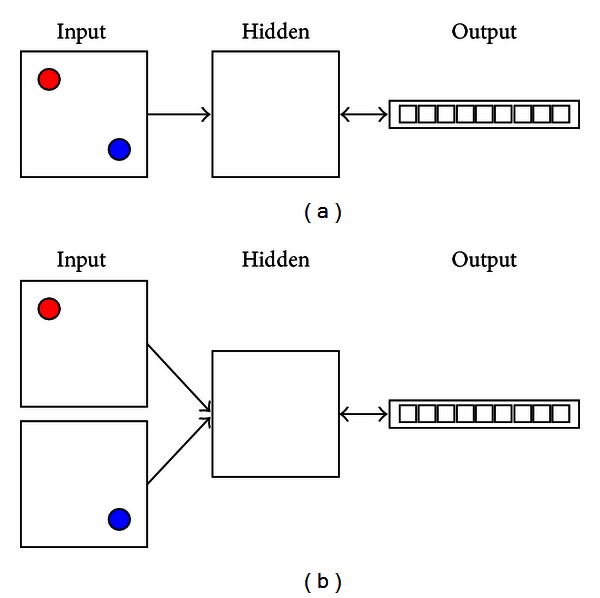
Two different configurations for providing the same spatial information to the PC module. (a) Two objects (e.g., a group center and a POI) are presented on the same input layer. (b) The same object pair is presented on two separate layers. When the task is to compute the Euclidean distance (flying distance) between the two objects, these two configurations yield indistinguishable performance. To compare the model performance, we computed the model-target correlation (correlation between the output values at the minus phase and the target values across trials). In both configurations, after training for 1000 epochs (20 trials in each epoch), the model can produce a model-target correlation greater than 0.95 in the last 10 epochs (*n* = 200 trials).

Another critical issue in organizing the input layers is to consider the principles of *perceptual segmentation* (e.g., to single out a particular set of objects from others) and *attentional foveation* (e.g., multiple scans in evaluating a large number of objects or estimating the distance across a wide range of visual field). We argue that separating a single enemy group from others (e.g., group attacks on PC_Attn) and representing a cluster of spatially distributed objects as a single object (group center on PCWM_GC) are essentially the results of these principles. The guideline is that such representations can be obtained and maintained in early visual processing, especially when the different groups of objects are displayed in different colors and can be easily distinguished from each other. Strong claims have been made based on the efficient detection of groups of image elements by selective neurons that occurs in higher areas of the visual cortex [33, 34]. Using a task of transsaccadic integration (TSI) in which participants used a mouse to click on the intersection point of two successively presented bars, Prime et al. [35] found indistinguishable performance in the “Saccade” condition (bars viewed in separate fixations) and the “Fixation” condition (bars viewed in one fixation) and concluded that participants can retain and integrate orientation and location information across saccades in a common eye-centered map in occipital cortex. From the perspective of attentional foveation, it is proposed that the dorsal stream (posterior parietal and lateral premotor cortices) plays the role of serial deployment of attention over different locations of space and/or time, such that the encoding of magnitude is abstract enough to respond to both sequential and simultaneous presentations [36, 37]. Together, the parietal cortex may receive multiple visual inputs in a rather flexible fashion. During our simulations, we have indeed found that different visual input formats can result in indistinguishable performances (Figure 5).

## 4. Output: A “ScalarVal” Representation of Magnitude

Currently, our PC module uses a “ScalarVal” type of Leabra layers to represent a magnitude value (“PC_Value” in Figure 3) [26, 38]. (For a detailed description of the ScalarVal specification, see http://grey.colorado.edu/emergent/index.php/ScalarValLayerSpec). Such a specification encodes and decodes scalar, real-numbered values based on a coarse coded distributed representation across multiple units (e.g., a value is represented by a Gaussian bump with a fixed standard deviation). This provides a very efficient and effective way of representing scalar values [39, 40].

**Figure 6:**
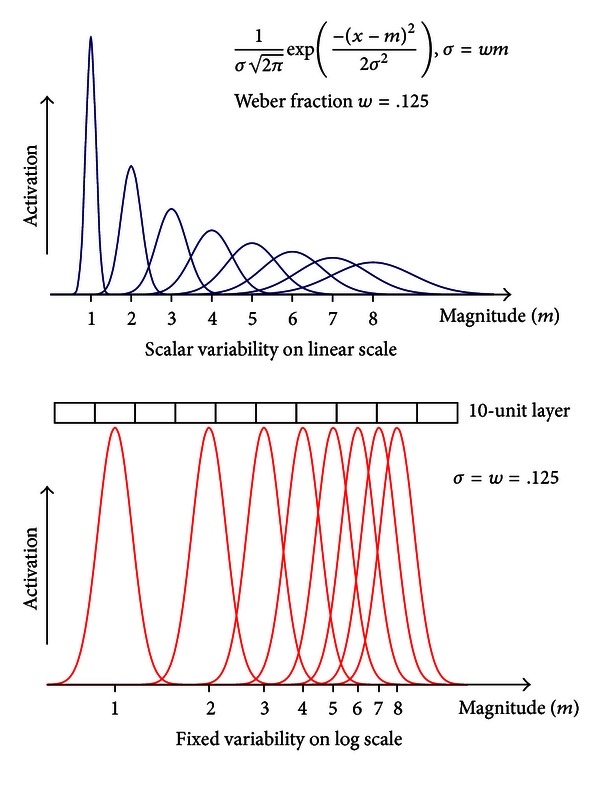
Comparison of the linear and logarithmic representation of magnitude. The method implemented in the PC module is analogous to the log scale representation in which we use a fixed number of units (thus fixed variance) to represent any particular level of magnitude.

On a related note, there has been an ongoing debate regarding whether magnitudes are being internally represented on a linear scale or a logarithmic scale (e.g., [41]) (see Figure 6). By linear encoding, the noise (i.e., standard deviation) in the internal representation of a magnitude is tied to the specific value of the physical magnitude. Then, in comparing two magnitudes *m*_1_ and *m*_2_, the discriminability (i.e., the amount of the overlap between two Gaussian distributions) is determined by the Weber fraction *w*, and the standard deviations that are tied to the specific values of the magnitudes (with a pooled standard deviation). By logarithmic encoding, the noise in the internal representation of *any* magnitude is solely determined by the Weber fraction. Discriminability is determined by the Weber fraction *w* and the ratio of two magnitudes *r* = *m*_1_/*m*_2_, regardless of the specific values of the magnitudes (Weber's law). In our opinion, the logarithmic encoding appears to be a more appealing candidate that makes the representation of a magnitude truly abstract and with generality. It should be noted that the linear and logarithmic representations are mathematically equivalent but have different advantages during actual computation (e.g., linear models are more convenient for addition and subtraction, and log models are more convenient for production and division). Because of the mathematical equivalence, it remains difficult to neurologically distinguish the actual representation form in the brain [42]. Nevertheless, the logarithmic representation appears to be more parsimonious in that the representation of a magnitude is independent of the range of the target values thus allowing different neurons representing different numbers to be activated in the same fashion (see Figure 6). In this regard, the default ScalarVal specification in Emergent serves our modeling purpose well.

## 5. Numerosity and Size on a Common Metric

The most significant aspect of the current PC module is that the computation of all types of target values (numerosity, radius, and two types of distances) largely shares a common pathway (see Figure 2). First, multiple input layers (e.g., individual group attacks, group center, POI, and road segment from direct visual and visual working memory slots) are projected onto different groups within a single hidden layer, depending on the particular task demand. This hidden layer employs a particular type of kWTA inhibition in that the winners are selected based on a combination of within-group and entire-layer inhibition (Figure 3). Its functions are analogous to those of the LIP area in that the spatial information is reencoded, *sensitively but not selectively* corresponding to the magnitude statistics from the visual environment. For example, a unit's activation may be statistically correlated with the number of active units on the input layer (i.e., sensitive to numerosity), but such a correlation on the hidden layer may not uniquely identify a number before being classified on the target layer (i.e., selectivity). In addition, the hidden layer receives a top-down signal from the layer “PFCcPC,” representing a single task demand for a particular type of target values (“PFCcPC” means “prefrontal controls parietal”). At the output level, the desired target value is represented on the single target layer “PC_Value,” analogous to the VIP area whose value representation *selectively* corresponds to the specific magnitude information in the visual environment. Computationally, the learning of target values occurs in two phases, an expectation-driven *minus phase* and an outcome-driven *plus phase* [26]. During the minus phase, the inputs (visual inputs plus the signal from PFCcPC) are reencoded onto the hidden layer and the target layer. During the plus phase, a teaching signal is provided on the target layer, which will provide a top-down correction by modifying the activations on the hidden layer and the corresponding weights. (For detailed descriptions of the learning rule (i.e., the extended contrastive attractor learning rule, XCAL), see http://grey.colorado.edu/CompCogNeuro/index.php/CCNBook/Learning).

The implementation of a common pathway for all types of target values is motivated by the following considerations. First of all, the recent literature suggests a “common metric” in parietal cortex responsible for the processing of all magnitude-like values such as numerosity, size, and temporal and spatial distances, namely, the temporal-spatial number line [5, 15, 43]. Second, although the projections from the input layers onto the various sections of the hidden layer are essentially in parallel, there is only one target layer. The rationale is that the perceptual stages operate in parallel but a central decision stage occurs via a serial bottleneck [44]. Third, the top-down control from PFCcPC reflects the idea that attention prioritizes stimulus processing on the basis of motivational relevance, and major sources of top-down attentional biasing have long been located principally in the dorsolateral prefrontal and posterior parietal cortices [45]. Also, the top-down connections from both PFCcPC and PC_Value to the hidden layer are consistent with the findings that the same neurons in LIP that encode values would also encode the selected actions late in the decision process [46].

Most importantly, the PC module addresses the dissociation and interference between various types of target values. Theoretically, there has been an ongoing debate regarding the interactions such as those between the processing pathways of numerosity, size, and density. On one side of the debate, it has been suggested that numerosity could only derive indirectly from texture density (e.g., [47–49]). On the other side, it has been suggested that numerosity could be an attribute “sensed directly” from the visual input, independently from texture perception [50, 51]. Most recently, Stoianov and Zorzi [52] shows that selectivity to visual numerosity emerges naturally during unsupervised learning in a hierarchical generative model of perception, invariant to area, density, and object features. This study has been cited by Ross and Burr [51] as a strong support to their theory of “visual sense of numbers.”

In the PC module, among the four types of target values, there are actually only two basic types of information being extracted from the visual environment: *numerosity* and *size*. As mentioned in the previous section, estimating the walking distance on a road segment is in effect a task of counting the number of active pixels. In addition, estimating the flying distance is in effect a size or radius estimation in which the number of objects is a constant of two, regardless whether the inputs are represented on a single or separate layers (see Figure 5).

**Figure 7:**
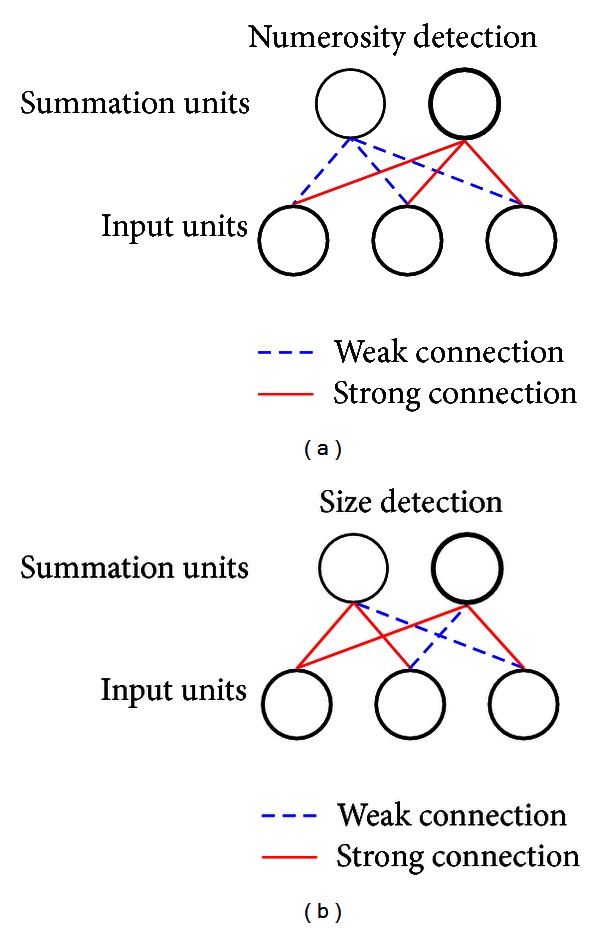
An illustration of numerosity detection (a) and size detection (b). In both figures, each unit on the hidden layer takes the sum of the visual input units, hence the name “*summation units*”. For simplicity, we show only three units as the visual inputs and two units on the hidden layer. In representing numerosity, a summation unit takes the sum from each visual input unit *uniformly* (i.e., with equal connection weights). Thus, the activation of such a unit only responds to numerosity monotonically, and the spatial information is completely discarded. Different summation units have different connection weights from visual inputs, and their combined activation pattern is projected to the final tuned numerosity detectors that are ultimately selective to numerosity. In contrast, the summation units for encoding size (or distance between the two furthest active visual units) must receive nonuniform weights *selectively* from different spatial locations in the visual inputs (i.e., with unequal connection weights) in order to preserve the spatial information.

It is important to note that the dissociation and interference of numerosity and size may occur at different levels of visual analyses. We hypothesize that the key in both of dissociation and interference lies in the mechanism in which neurons selectively or uniformly sample the visual field and whether the spatial information is discarded or preserved during the sampling (see Figure 7). In the case of numerosity representation, it has been found that there were “summation units” in the parietal lobe, particularly in the LIP area, whose responses resembled the output of accumulator neurons that systematically increased or decreased with the increase of the numerosity in visual stimulus [53]. And, there were “number neurons” tuned to a preferred numerosity with “labeled-line” encoding of numerosity in the VIP area [54–57]. Thus, similar to some of the previous models on numerosity [52, 58, 59], our approach to modeling both numerosity and size estimation is to assume that the final tuned magnitude detectors on the target layer “harvest” the activations from the preceding summation units on the hidden layer. In order to selectively respond to the numerosity information, the spatial information must be discarded (e.g., the number sense of “2” arises regardless how far two objects are apart from each other). One immediate way to achieve such a dissociation is to assume the numerosity summation units samples the visual field uniformly (with approximately equal connection weights), regardless the spatial locations (see Figure 7(a)). This kind of uniform sampling has been demonstrated by [59]. On the other hand, spatial location information has to be preserved in size detection, which implies that the summation units must selectively cover different locations in their receptive fields (see Figure 7(b)).

What is interesting is how the interference between numerosity and size can arise when the spatial location information is only partially discarded or preserved. It has been found that single neurons tuned to quantity can provide information about only a restricted range of magnitudes, and only the population of selective neurons together can account for the entire range of tested stimuli [60]. Thus, it is likely that the receptive field of individual *numerosity summation units* on the hidden layer is spatially segmented and they are selective to a limited region of space, especially in a high-load condition (e.g., when the scene is crowded or subjects are distracted). Otherwise, responding to a greater range of numerosity would require finer graded activation levels thus overburden the summation neurons. As a result of the spatial segmentation, the activation of these neurons would partially carry the location information from the visual inputs. On the other hand, some of the *size summation units* may take visual inputs less selectively regarding different spatial locations. In either scenario, we would expect that the numerosity-size dissociation by the summation units is not perfect (i.e., carrying partial spatial information) and observe some neurons serving a double duty on both numerosity and size detection. We can find support to such a speculation repeatedly from both neurological (e.g., [5, 8, 54] and behavioral studies [48, 49]). Particularly, in their transcranial magnetic stimulation (TMS) experiments, Kadosh et al. [61] have found that the interference between number and size is late in the processing stream, at the point of response initiation and interaction between the stimulus attributes only in high-load conditions. And, it has been proposed that the numerosity and size estimations, and their overlaps, arise as the results of the serial deployment of attention over different locations of space and/or time via the dorsal stream (posterior parietal and lateral premotor cortices) [36, 37].

## 6. Topological Comparison and Representativeness

Although the main scheme in our PC module is metric estimations in a serial fashion (i.e., only one type of metrics is available at a time at the output level; see Figure 2), it should be emphasized that some bottom-up processing may indeed have occurred in a parallel fashion, resulting in behaviorally relevant representations in the process of decision making. In particular, the global nature of perceptual organization of spatial information has been described in terms of topological invariants, prior to the perception of other featural properties; that is, the processing of topological information may occur earlier than any metric estimation (e.g., [62]). Moreover, It has been suggested that posterior parietal cortex (PPC) supports topological spatial information which emphasizes the importance of proximity of local landmark cues, whereas the hippocampus supports metric spatial information which emphasizes the importance of distance between local landmark cues [25, 63, 64].

Whereas the metric information is defined as the relationship of angles and distances between objects resulting in a continuous representation of values (e.g., radius and distances in the COIN-AHA problems), the topological relationships are represented by a connectedness relationship between objects that are invariant of metric modifications resulting in a categorical representation of values [25]. If topological comparisons indeed have occurred earlier than metric estimation, it would be very plausible that they are utilized in the decision-making process, especially as the means of shortcuts in the early stages. For example, it has been reported that expert geographers organized their thoughts and presented data to others with the topological information [65–67]. 

**Figure 8:**
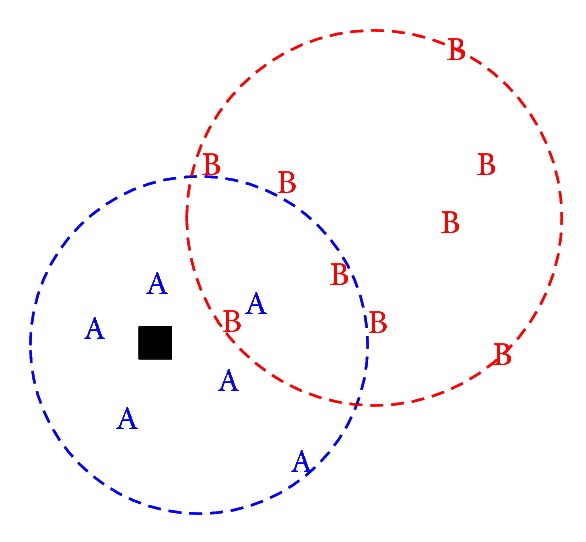
Topological comparison in an example trial in COIN-AHA task. The point of interest (POI, the black square) falls *inside* the region of Group A's attacks, but *outside* the region of Group B's attacks. Thus, without estimating the more abstract spatial information such as distance and numerosity, the POI might be perceived as more representative of the spatial characteristics of Group A's attacks than that of Group B.

Perhaps more significantly, modeling the topological comparison would enable us to examine the *representativeness heuristic* that might arise at the level of perceptual analysis (Figure 8). It should be noted that the term of representativeness heuristic has been coined more than three decades ago [68]. Here we take a more updated interpretation described by Kahneman and Frederick [69]. According to this interpretation, both of the representativeness and availability heuristics in effect belong to the heuristic of accessibility and substitution, where an individual assesses a specified target attribute of a judgment object by substituting another property of that object—the heuristic attribute—which comes more readily to mind. Applied to the COIN-AHA tasks, it is possible that when human subjects perceive that the POI falls inside the region of Group A but outside the region of Group B, they might conclude that this particular POI is more representative of Group A's characteristics than that of Group B. Consequently, they might draw a conclusion that Group A is more likely to be responsible for the attack. That is, a decision can be made by taking a shortcut where the topological relation is used as a heuristic attribute to substitute a metric estimate of distance which only arrives later. 

## 7. Module Performance

The PC module was trained within the integrated model with artificial data generated by a data generation software. (For details of the integrated model, see, [70]). At the current stage, we only focused on the training on metric estimates (e.g., numerosity, radius, flying distance, and walking distance). The training on topological comparison has not been completed thus it is omitted here. In addition, it has been found that in the COIN-AHA tasks, human subjects have mainly relied on the metric distances as the predictor [71]. 

**Figure 9:**
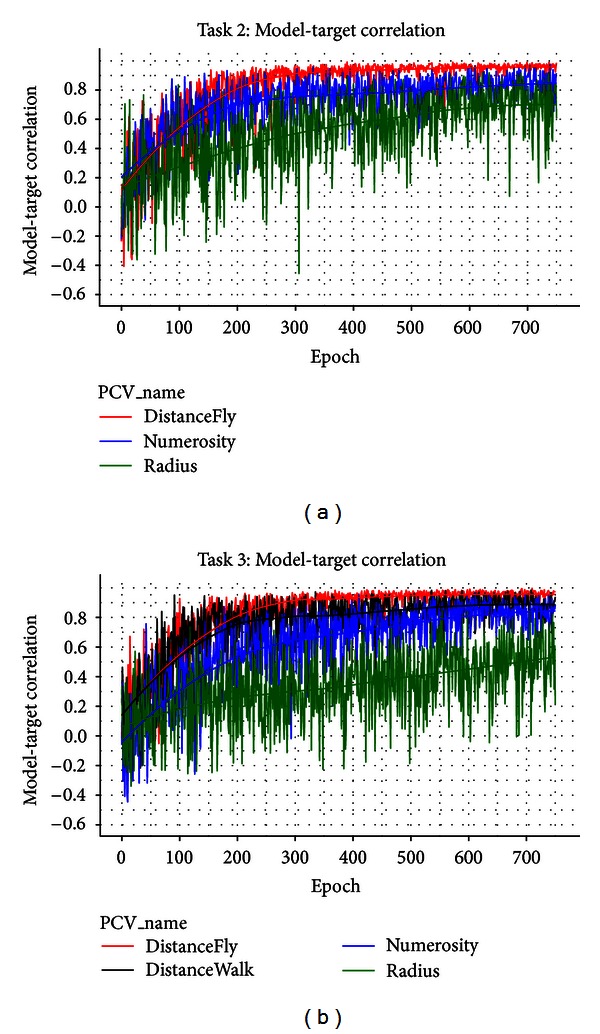
Performance of the PC module in Task 2 (a) and Task 3 (b) measured by model-target correlation. In both figures, the top-down PFCcPC-to-Hidden connections are specified as “group-one-to-one.” In such a connection, each unit on PFCcPC is connected to all units in the corresponding section on the hidden layer but not other sections. For example, the unit on PFCcPC representing “numerosity” is connected with all units in the bottom-right quarter on the hidden layer (see Figure 3). Task 2 does not have a road network so that no training on DistanceWalk.

Overall, we have demonstrated that the PC module can accurately extract various types of target values from the training dataset. To measure the module performance, we use the model-target correlation, which is the correlation between the minus phase activation on the target layer (“PC_Value”) and the corresponding target value across trials within each epoch.  Figure 9 shows the module performance in COIN-AHA Task 2 (without road network) and Task 3 (with road network). It can be seen that, in both tasks, the performances on Numerosity, DistanceFly, and DistanceWalk were very accurate (model-target correlations greater than 0.8 after 750 epochs). The only difference is that the training on Radius in Task 3 only showed a moderate performance. It is noted that the performance on DistanceFly was consistently more accurate than the performances on DistanceWalk and Numerosity. One apparent reason is due to the different levels of variances and ranges of the target values that are embedded in the input representations. For example, on a 24 by 24 grid, the maximum distance on the diagonal is 24 × 1.414 *≈* 34, but the maximum DistanceWalk and Numerosity can be 24 × 24 = 576. The moderate performance on Radius can also be attributed to the variance on the input representations in which the location changes of individual units may not change the overall dispersion but can significantly affect the spatial correlations between units. That is, unlike other target values, the interactions between units can add additional noises in the model performance on Radius. 

Importantly, Figure 9 shows the dissociation among various types of target values. In particular, the model-target correlations for both Numerosity and Radius in Task 2 reached approximately 0.8 after 750 epochs. Given that both target values have to be computed from the same visual input on PC_Attn, such a performance suggests an almost perfect dissociation between Numerosity and Radius (i.e., size). Crucially, this result has been obtained with the specific top-down connections from PFCcPC to the hidden layer (see Figure 3). That is, the units on PFC, each representing a unique demand, are, respectively, connected to the corresponding sections on the hidden layer. For example, the unit on PFCcPC representing “numerosity” is connected with all units in the “numerosity” section on the hidden layer, but not the units in other sections. As a result, when the demand from PFCcPC is to compute “Numerosity”, the corresponding section of the hidden layer is more likely to be activated thus wins the inhibition competition over other sections. In other words, the top-down signal from PFCcPC provides a critical role in the functionality specialization on the hidden layer.

**Figure 10:**
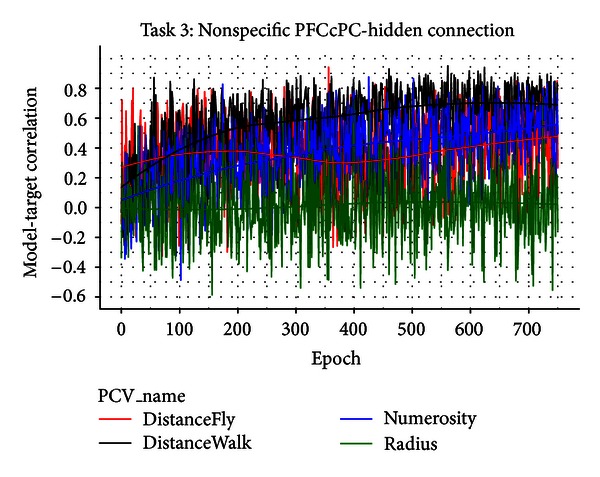
Performance of the PC module in task 3, with nonspecific PFCcPC-to-Hidden connections (e.g., each unit on PFCcPC is connected with all units on the hidden layer). It appears that the lack of specificity in the top-down control has caused the module's failure in dissociating between different target values.

In contrast, Figure 10 shows the module performance in Task 3 with nonspecific PFCcPC-to-Hidden connections (e.g., each unit on PFCcPC is connected with all units on the hidden layer). It can be seen that dissociation had occurred to some extent, but the model-target correlation has significantly dropped for all types of target values. For example, the highest model-target correlation in Figure 10 was achieved on DistanceWalk, 0.65 (*n* = 100 trials), which was significantly lower than that in Figure 9, 0.86 (*n* = 100 trials) (comparing the two correlations by Fisher *r*-to-*z* transformation, *Z* = −3.61, two tailed *P* < .001). Since the only difference in these results was in the way PFCcPC is connected to the hidden layer, it appears that the failed dissociation between different target values is due to the lack of specificity in the top-down control. This finding is consistent with the current understanding in the literature on selective attention. For example, it has been suggested that the goal-directed attention can prioritize stimulus processing on the basis of motivational relevance via the dorsolateral prefrontal and posterior parietal network [45]. In our model, the top-down control was implemented by the PFCcPC-to-Hidden connections. With the specific connections, the active unit on PFCcPC is only projected onto the corresponding section on the Hidden layer. As a consequence, the units on that section are more likely to be active and better associated with the current target value since the corresponding connection weights are updated based on the activation values. Thus, by a goal-directed division of labor, different groups of units on the Hidden layer can develop associations with their own target values in a relatively independent fashion, resulting in an overall better performance. 

**Figure 11:**
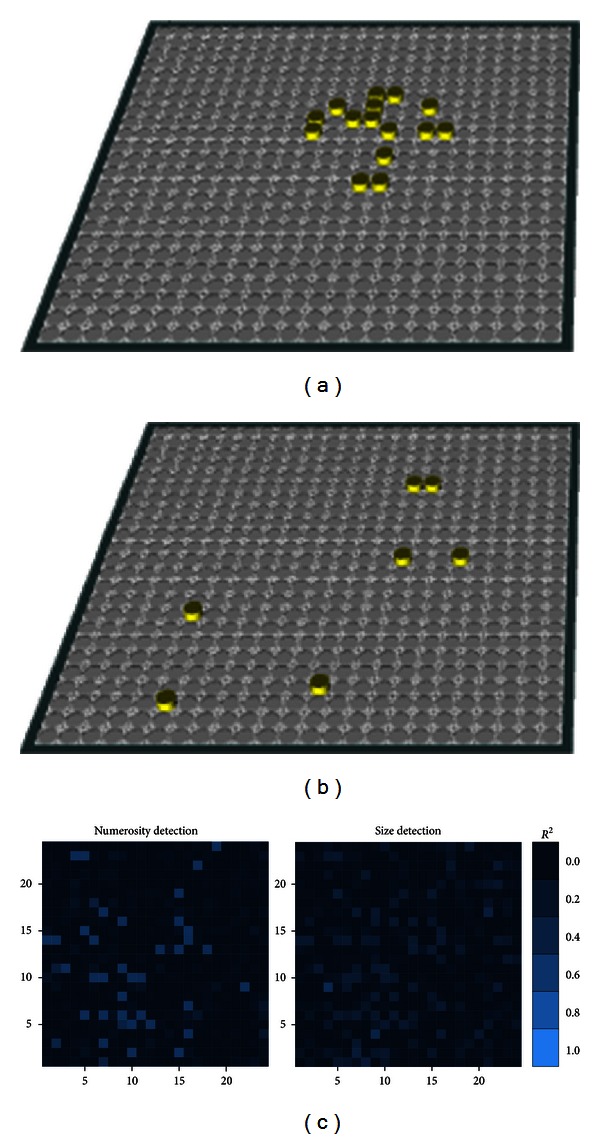
The dissociation and interference between numerosity and size. (a) A visual input with high numerosity but small patch size. (b) A visual input with low numerosity but large patch-size. (c) Without top-down control and teaching signals, units on the same hidden layer show sensitivity to either numerosity (left panel) or size (right panel), or both (the overlap of unit locations). Sensitivity is measured by *R* ^2^ in a linear regression of the activation on the respective magnitude.

Besides attention, expectation is considered as another top-down mechanism that mitigates the burdens of computational capacity in visual cognition, which may lie more medially in the posterior cortices as well as more ventrally in the frontal lobe [45]. In the PC module, the top-down control is not only from PFCcPC but also from the teaching signals provided on the target layer. As mentioned in the early section, it has been debated whether numerosity is a property that can be “sensed directly” from the visual input, dissociated from texture perception [51, 52]. To test this idea, we also conducted simulations with a simplified PC module with only one visual input layer and one hidden layer, and without teaching signals and top-down signals from PFCcPC (Figure 11). We find that by pure Hebbian association, units on the hidden layer can indeed show some dissociation between numerosity and size, but only to a certain extent. First, the correlation between hidden unit activation and either target value was hardly perfect (similar to the findings by [52]). For example, when computing the model-target correlations over 100 trials, a Pearson product-moment correlation coefficient of merely .254 can reach statistical significance level *P* < .01. Thus, a unit could be classified as a “numerosity neuron” without being able to selectively identify specific numbers. Second, many units showed overlapped sensitivity to both numerosity and size. That is, our finding appears to be more consistent with the proposal by Dakin et al. [48] that people's senses of number and density are intertwined (note that density = numerosity/size). Combined with the results shown above (e.g., Figures 9 and 10), it appears that perfect dissociation between numerosity and size can indeed occur, provided that there are specific goal-directed top-down controls.

## 8. Discussion

In this paper, we describe an integrated model of the parietal cortex for spatial-temporal information processing in sensemaking. In summary, the development of the PC module suggests that, with quite similar structures, different types of environmental statistics (e.g., numerosity, size, Euclidean distance between two points, and length of curved line segment) can be extracted from visual inputs then represented as a magnitude value, supporting the proposal of a “common metric” housed in the parietal cortex (e.g., [5]).

The most significant finding from our simulations is that although early visual dissociation can occur between different types of environmental statistics, the goal-directed top-down control appears to be critical towards a complete dissociation. This finding is consistent with the current understanding in the literature on selective attention. In our model, the top-down control was implemented by the PFCcPC-to-Hidden connections. We demonstrated that high model-target correlations could be achieved only when the connections are specified with particular top-down projections. The interference without specific top-down controls can be more easily understood regarding how the kWTA inhibition mechanism would affect the dissociation between different types of environmental statistics. Crucially, different types of environmental statistics are obtained at different levels of abstraction. For example, a completely accurate estimation of numerosity (DistanceWalk and Numerosity) requires a complete spatial invariance whereas estimation of Euclidean distance (DistanceFly) or dispersion (Radius) is essentially based on spatial correlation. By the randomly initialized weights, some units may be biased towards a certain level of spatial invariance. However, with the kWTA inhibition in place, only the most active units have the chance to be updated and associated with the current target value. Thus, even when some units have shown some sensitivity to a certain type of target statistics at the early perceptual stage, such sensitivity may not be able to propagate further for the selectivity to be developed. One direct way to neurologically corroborate our simulation findings is to examine whether the task demand from the learning environment can interfere with the roles of neurons, for example, causing neurons initially sensitive to numerosity to be sensitive to size. 

Moreover, whereas our current model focuses on the interference and dissociation of different types of spatial information within the parietal cortex, it is also possible that other cortical topologies and mechanisms may contribute to the similar process. For example, it has been suggested that the mosaic organization of the superficial layers of the dorsocaudal medial entorhinal cortex (dMEC) represents a possible substrate for the modularity of the spatial map, which is an indication of early dissociation of different types of spatial information [72]. In addition, our current emphasis in modeling the parietal cortex is on the dissociation and representation of magnitude values, and a major goal is to reduce interference thus achieve high accuracy in performance. Accordingly, we have made several simplifications in modeling many of the subtasks. For example, we did not distinguish the processes of exact counting and subitizing (when the enumeration of objects is fast and accurate for sets of up to three or four items) [36]. And, we have avoided the problem of finding the shortest path in estimating the walking distance along the road.

In general, our modeling effort attempts to strike a balance between two types of preferences: whether to emphasize the mechanism of “attentional foveation” or to emphasize the mechanism of “perceptual segmentation” and “topological grouping.” The former requires multiple sequential representations in the model (e.g., multiple scans in a crowded scene, exploration of all road segments between two points), and the latter makes it plausible to represent a set of stimuli as a whole (e.g., multiple objects of the same color segmented from others, a single road segment between two points). For example, the “zoom lens model” postulates that curve tracing has to be carried out in multiple passes each with a different foveation [73]. That is, in estimating the walking distance along the road, it would involve scanning multiple road segments between multiple intersections and points of interests and estimating distances according to different reference points. Neurologically, it has been posited that the parietal cortex is responsible for the transition between reference systems (e.g., [24, 74]). From behavioral studies, we have argued that the selection of reference systems (e.g., egocentric versus intrinsic) is an essential component in the internal representation of physical distances and relative locations [21, 23]. Thus, implementing the mechanisms of attentional foveation and selection of reference systems would lead to a more realistic model with the ability to identify some of the human heuristics and biases in spatial representation and reasoning. In the current model, all of the visual information is presented on the input layers at once. Thus, the model completely lacked the mechanism of attentional foveation. In addition, the selection of reference systems was rather fixed such that the model lacked the mechanism of flexibly changing the anchors of the reference system. We will further pursue these potential improvements in future research.
